# Sjogren's Syndrome and Clinical Benefits of Low-Dose Naltrexone Therapy: Additional Case Reports

**DOI:** 10.7759/cureus.8948

**Published:** 2020-07-01

**Authors:** Scott Zashin

**Affiliations:** 1 Rheumatology, University of Texas Southwest Medical Center, Dallas, USA

**Keywords:** sjogren syndrome, low dose naltrexone, joint pain, fatigue, autoimmune diseases

## Abstract

Sjogren’s syndrome (SS) is a chronic autoimmune disorder that causes the inflammation of the lacrimal and salivary glands, resulting in dryness of the eyes and mouth. In addition, fatigue and musculoskeletal pain, often described as aching, are very common. Treatment directed towards alleviating the fatigue and pain associated with SS is currently very limited. In March of 2019, the first peer reviewed case report showing clinical improvement using low-dose naltrexone (LDN) in a patient with suspected SS was published in Cureus. This report describes two additional patients with SS whose conditions responded favorably to a treatment with LDN therapy. The first case is a 24-year-old female with documented SS. Her diagnosis was based on a history of dry eyes, dry mouth, joint pain, fatigue, and headache. In addition, she had very high measures of inflammation and a positive anti SS-A antibody. She improved clinically with LDN therapy. The second case is a 66-year-old female with documented SS based on a history of dry eyes and dry mouth, joint pain, and elevated anti-SSA and anti-SSB antibodies whose joint symptoms responded to treatment with LDN.

## Introduction

Low-dose naltrexone (LDN) is a unique compound that has pain-relieving and anti-inflammatory properties. Limited studies have shown benefit in helping relieve the pain in patients with fibromyalgia and improving disease activity in autoimmune conditions such as inflammatory bowel disease and multiple sclerosis. A case report published last year demonstrated effectiveness of LDN in a patient with Sjogren's syndrome (SS) based on improvement of their symptoms and inflammatory markers. Two additional cases of SS presented in this article demonstrated similar efficacy in clinical symptoms and lowering of inflammatory markers.

## Case presentation

Case one

BB is a 66-year-old female seen who carried a diagnosis of SS. The diagnosis was based on a history of joint pain without synovitis or tenderness, dry eyes, and dry mouth. She had a positive antinuclear antibody (ANA) 1/160 in a speckled pattern; positive anti-SS-A and SS-B at 418 and 180 U/mL respectively (normal less than 100 U/mL). Her rheumatoid factor (RF) was 175 IU/mL (normal less than 10 IU/mL). Some 18 months prior to her first visit she had started plaquenil orally at 200 mg per day, with improvement in her joint symptoms. On her initial evaluation, she felt well and her erythrocyte sedimentation rate (ESR) was 12 mm/h and C-reactive protein (CRP) was 0.16 mg/dL (normal less than 0.80 mg/dL).

The patient reported that she was feeling well. About eight years ago, she was concerned about the risk of retinal toxicity from plaquenil, so she elected to decrease the dose to 100 mg daily. About five years ago, she stopped plaquenil completely as she felt well. Within four months of discontinuing the drug she developed mild joint pain and an increase in her ESR to 29 mm/h. About three years ago, due to increasing pain she resumed plaquenil 200 mg daily. However, her symptoms persisted and her sedimentation rates remained elevated between 27 and 46 mm/h. Over the past year, her symptoms increased and were associated with bilateral synovitis in two metacarpophalangeal (MCP) and two proximal interphalangeal (PIP) joints. In addition, there was an associated increase in her inflammatory markers with a CRP of 11.3 mg/L. Her sedimentation rate at that time was 35 mm/h. She elected to try LDN after improvement with a one-week course of prednisone. One month after being on 1 mg of LDN, she noted less pain and inflammation. Her ESR went down to 28 mm/h and CRP normalized at 5.7 mg/L. Four months ago, she had increased her LDN to 2 mg daily. She felt well clinically without any joint pain. Her exam failed to show any hand tenderness or swelling. While her ESR remained high at 40 mm/h, her CRP was down to 4.6 mg/L. As of one month ago, she remained asymptomatic with a normal ESR of 23 mm/h and a normal CRP of 2.1 mg/L.

Case two

CC is a 24-year-old female first evaluated in November of 2018. She noted a five-year history of chronic widespread body pain, fatigue, headaches, and brain fog. Her previous lab showed a negative ANA, an increased ESR of 40 mm/h, and an increased CRP of 7.8 mg/L (normal less than 5 mg/L). She continued to be symptomatic, despite a gluten free diet, physical therapy, and antidepressants. Her medications included IV gamma globulin. Hydrocodone 10/325 5 tabs daily, flexeril qhs, benadryl daily, metformin, topiramate, baby aspirin, and metoprolol. She noted dry eyes and dry mouth. Her examination confirmed widespread myofascial pain in four quadrants without synovitis. Lab work showed a positive anti-SSA antibody 42 U/mL (normal less than 7 ELISA method). Her ANA was positive at 1/80 homogeneous and her other specific lupus and rheumatoid markers were normal. Her ESR was quite elevated at 90 mm/h and CRP 14.2 mg/L (normal less than 8.0 mg/L). Her globulin was 4.8 g/dL (normal less than 3.7 g/dL). She was started on LDN 0.5 mg po daily which was increased weekly up to a target dose of 4.5 mg. Five weeks after starting LDN she was currently taking 2.5 mg daily. Her ESR and CRP improved to 42 mm/h and 6.8 mg/L respectively. She stated her joint pain and headache were significantly better. Some six months after starting LDN she was currently taking 4.5 mg (had been on this dosage for three months) and she continued to feel well but noted fatigue. She requested a prescription for plaquenil, but she never started the medication. Instead, it was elected to increase the LDN gradually over the next two months to 8.5 mg. One year after starting LDN, her headaches were better, but she did have persistent pain which corresponded to trigger points on the examination consistent with fibromyalgia. The patient complained of fatigue. Despite these symptoms her CRP was now normal for the first time at 3.5 mg/L and her ESR was also normal at 16 mm/h suggesting an improvement in the inflammatory aspect of her condition.

## Discussion

In the initial pilot study that used LDN in the treatment of fibromyalgia, the baseline sedimentation rate was a significant predictor of clinical response to LDN. It was of interest to note that in the two case presentations above, the patients’ clinical response to LDN correlated with an improvement in ESR. Dosing and purported mechanism of action is discussed in detail elsewhere [1-2]. Based on these case reports, both patients experienced significant clinical improvement in their musculoskeletal issues which include arthralgia, a common symptom in patients with SS. One patient also had improvement in fatigue which is present in most patients with this autoimmune disease and can be debilitating. On the other hand, dry eyes and dry mouth which are cardinal features in the diagnosis of SS did not improve with the use of naltrexone in these two patients. Due to the lack of FDA approved treatments for the constitutional features of SS and the low cost as well as favorable safety profile of LDN, this medication can be very beneficial in improving the quality of life for those patients afflicted with this disorder.

## Conclusions

This report describes two additional cases of patients with active SS who were treated with LDN and obtained clinical benefits. As a result of these case studies, further research consisting of a double blind controlled trial is needed to determine if LDN will subsequently prove to be a useful medication for treating SS. Until then, it appears that LDN may be a useful therapy to help with joint pain and other constitutional symptoms in patients with this disorder.

## References

[1] Zashin SJ (2019). Sjogren's syndrome: clinical benefits of low-dose naltrexone therapy. Cureus.

[2] Younger J, Mackey S (2009). Fibromyalgia symptoms are reduced by low-dose naltrexone: a pilot study. Pain Med.

